# Supplementary material for: MetaRibo-Seq measures translation in microbiomes
Source: Nat Commun. 2020 Jun 29;11:3268. doi: 10.1038/s41467-020-17081-z (PMC7324362; doi:10.1038/s41467-020-17081-z)
Supplement: Supplementary file 10 — Supplementary Data 7 [file 41467_2020_17081_MOESM10_ESM.zip › File2/Confidence_High_Taxonomy/209789_out.krona.html]

Javascript must be enabled to view this page.

members
magnitude
magnitudeUnassigned
count
unassigned
taxon
rank

209789\_out

94

superkingdom
2
94

94
976
phylum

class
200643
94

order
171549
94

family
815
93

816
93
genus


SRS021948\_contig\_number\_contig-100\_1002.179602SRS023526\_contig\_number\_10150SRS063813\_contig\_number\_contig-100\_73.57304SRS142503\_contig\_number\_38657SRS147346\_contig\_number\_12506
species
818
5


SRS014613\_contig\_number\_19845SRS064276\_contig\_number\_40794
species
2292935
2

1
291645
species

SRS046717\_contig\_number\_5529


SRS017433\_contig\_number\_9441SRS053398\_contig\_number\_1860SRS078665\_contig\_number\_1903SRS105153\_contig\_number\_2377SRS893341\_contig\_number\_1035
species
46506
5

2292956
2
species

SRS014613\_contig\_number\_8303SRS018427\_contig\_number\_14455


SRS075878\_contig\_number\_14950
species
1263048
1

8
457391

SRS013215\_contig\_number\_625SRS020233\_contig\_number\_41845SRS063985\_contig\_number\_22685SRS077086\_contig\_number\_contig-100\_768.78035SRS148721\_contig\_number\_25339SRS149879\_contig\_number\_contig-100\_410.146490SRS893300\_contig\_number\_8868SRS893342\_contig\_number\_contig-100\_813.62921
species

species

SRS147425\_contig\_number\_132
1
387090

species

SRS105153\_contig\_number\_27048
1
47678

species

SRS013800\_contig\_number\_1987SRS016056\_contig\_number\_5402SRS016954\_contig\_number\_11395SRS020328\_contig\_number\_16190SRS047433\_contig\_number\_18921SRS049446\_contig\_number\_6625SRS050026\_contig\_number\_12134SRS050941\_contig\_number\_8020SRS058770\_contig\_number\_9497SRS064645\_contig\_number\_1211SRS078242\_contig\_number\_28547SRS100021\_contig\_number\_10710SRS1041139\_contig\_number\_1408SRS143895\_contig\_number\_41590SRS892882\_contig\_number\_10089
2292944
15


SRS016018\_contig\_number\_1256SRS023346\_contig\_number\_760SRS024663\_contig\_number\_8305SRS050925\_contig\_number\_34636SRS1054691\_contig\_number\_673SRS140645\_contig\_number\_2163
species
357276
6

1262737
1
species

SRS012969\_contig\_number\_24394

329854
1

SRS1055099\_contig\_number\_9877
species

species

SRS1041134\_contig\_number\_contig-100\_428.67573
1
1739298

246787
1

SRS024331\_contig\_number\_673
species

1
671267

SRS1041132\_contig\_number\_1188
species


SRS015960\_contig\_number\_17432
species
1
820


SRS015890\_contig\_number\_368
species
1
162156

species

SRS014459\_contig\_number\_31225
310298
1

species

SRS893285\_contig\_number\_74
1
310297


SRS015579\_contig\_number\_24825SRS017521\_contig\_number\_39430SRS023971\_contig\_number\_contig-100\_27.38636SRS049900\_contig\_number\_10822SRS053398\_contig\_number\_18560SRS055966\_contig\_number\_contig-100\_1560.24562SRS063370\_contig\_number\_10059SRS104636\_contig\_number\_20225SRS893383\_contig\_number\_20744
species
9
817

species

SRS014235\_contig\_number\_35011SRS015217\_contig\_number\_27441SRS017375\_contig\_number\_643SRS020394\_contig\_number\_7329SRS103987\_contig\_number\_16515SRS1041036\_contig\_number\_4425SRS1041091\_contig\_number\_12291SRS1041144\_contig\_number\_4578SRS1041147\_contig\_number\_1057
9
204516

species

SRS014736\_contig\_number\_2760SRS016095\_contig\_number\_27700SRS018888\_contig\_number\_9372SRS019601\_contig\_number\_27916SRS046068\_contig\_number\_574SRS053356\_contig\_number\_37770SRS078419\_contig\_number\_24405SRS098655\_contig\_number\_1621SRS1041031\_contig\_number\_27089SRS104327\_contig\_number\_6276SRS142712\_contig\_number\_3918SRS144297\_contig\_number\_12529SRS144603\_contig\_number\_11348SRS148784\_contig\_number\_13799SRS149784\_contig\_number\_contig-100\_176.162587SRS149879\_contig\_number\_23650SRS893380\_contig\_number\_912
821
17

2292939
1
species

SRS101376\_contig\_number\_25347


SRS893369\_contig\_number\_8619
species
1
2292941

2005525
1
family

genus
1
375288

46503
1

SRS1055095\_contig\_number\_contig-100\_814.815
species
